# Supplementary material for: Towards Probing Conformational States of Y2 Receptor Using Hyperpolarized 129Xe NMR
Source: Molecules. 2023 Feb 2;28(3):1424. doi: 10.3390/molecules28031424 (PMC9919357; doi:10.3390/molecules28031424)
Supplement: Supplementary file 1 [file molecules-28-01424-s001.zip › molecules-2139581-supplementary.pdf]

SUPPLEMENTARY MATERIALS

# Towards Probing Conformational States of Y2 Receptor Using Hyperpolarized $^{129}$Xe NMR

**Peter Schmidt [1,†], Alexander Vogel [1,†], Benedikt Schwarze [1], Florian Seufert [1], Kai Licha [2], Virginia Wycisk [2], Wolfgang Kilian [3], Peter W. Hildebrand [1] and Lorenz Mitschang [3,*]**

[1] Institute of Medical Physics and Biophysics, Medical Faculty, University of Leipzig, Haertelstrasse 16-18, 04107 Leipzig, Germany
[2] Institute of Chemistry and Biochemistry, Freie Universitaet Berlin, Takustrasse 3, 14195 Berlin, Germany
[3] Physikalisch-Technische Bundesanstalt Braunschweig und Berlin (PTB), Abbestrasse 2-12, 10587 Berlin, Germany
* Correspondence: lorenz.mitschang@ptb.de
† These authors contributed equally to this work.

# SUPPLEMENTARY MATERIALS CONTENT

**Figure S1:** Plot of the different contact probabilities

**Figure S2:** Plot of RMSD values over simulation time

**Table S1.** List of the contact probabilities of Y2R

**Table S2.** List of the contact probabilities of NPY

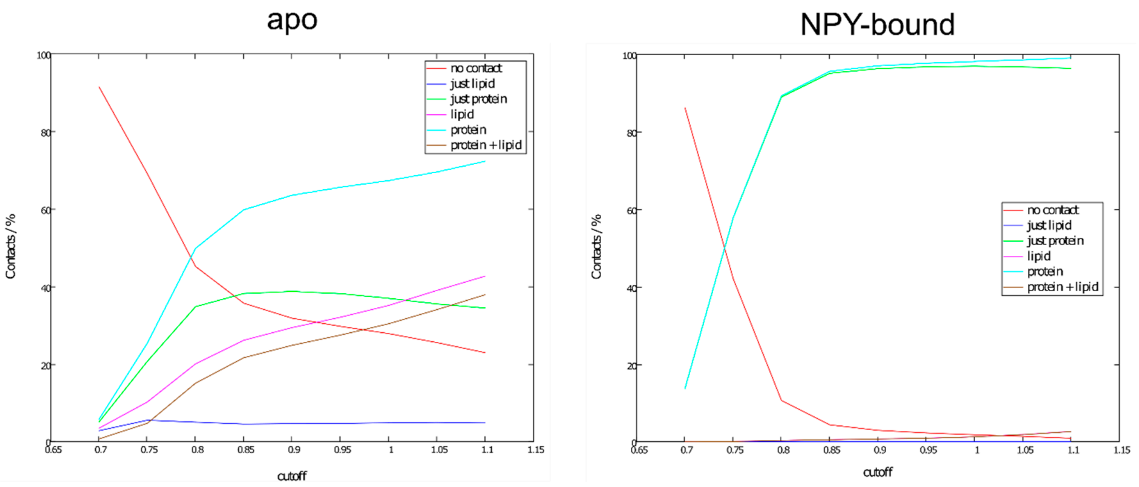


**Figure S1.** Plot of the different contact probabilities vs. the choice of cutoff in nm. From this figure we concluded that 0.85 nm is a good choice for the cutoff, where we have good separation of the different types of contact. A larger cutoff would lead to having contact to many different types of simulation constituents at the same time and a smaller cutoff would lead to very few contacts at all. In particular from the line “no contact” in the NPY-bound case it is clear that a cutoff of 0.85 nm is sufficient to capture almost all contacts.

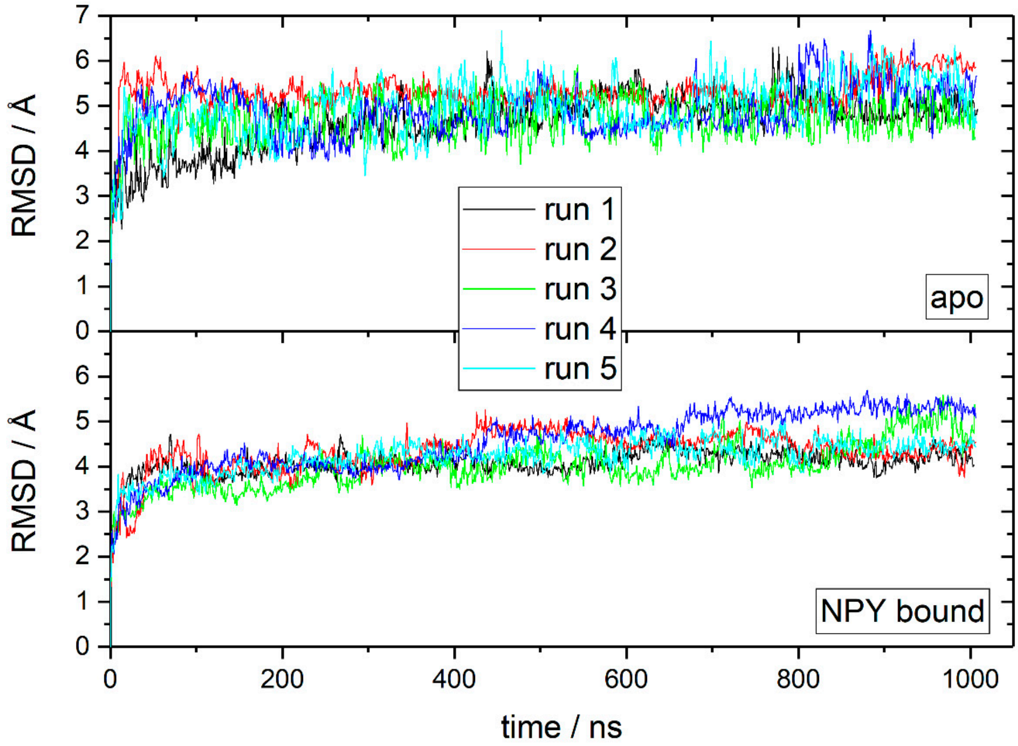


**Figure S2.** Plot of RMSD values over simulation time for run 1 through 5 (out of 30) for both systems in presence of the cage. For both systems RMSD values are below 7 Angstroem the whole time. Values are slightly higher for the apo system in general. This is to be expected since this system was started from a Y2R structure where NPY (and the G-proteins) were present and have been removed. This resulted in an empty ligand binding pocket, that was open and therefore easily accessible to the solvent and to the cage as well. This was intentional to check if the cage with the Xenon atom inside was able to penetrate the ligand binding pocket in the apo state. Therefore, this structure is slowly approaching the deactivated state, which is the reason for the slightly higher RMSD values.

**Table S1.** List of the contact probabilities with Xenon for each individual amino acid of Y2R in the apo state and in the NPY-bound state.

| Y2R amino acid | 40 | 41 | 42 | 43 | 44 | 45 | 46 |
|---|---|---|---|---|---|---|---|
| apo Y2R | 0.1760 | 0.1448 | 0.0365 | 0.0280 | 0.0463 | 0.0919 | 0.0595 |
| NPY-bound Y2R | 0.1949 | 0.0670 | 0.0262 | 0.0251 | 0.0114 | 0.0017 | 0.0004 |

| 47 | 48 | 49 | 50 | 51 | 52 | 53 | 54 | 55 |
|---|---|---|---|---|---|---|---|---|
| 0.0918 | 0.0131 | 0.0024 | 0.0133 | 0.0333 | 0.0000 | 0.0000 | 0.0021 | 0.0003 |
| 0.0000 | 0.0000 | 0.0000 | 0.0000 | 0.0000 | 0.0000 | 0.0000 | 0.0000 | 0.0000 |

| 56 | 57 | 58 | 59 | 60 | 61 | 62 | 63 | 64 |
|---|---|---|---|---|---|---|---|---|
| 0.0000 | 0.0000 | 0.0000 | 0.0000 | 0.0000 | 0.0000 | 0.0000 | 0.0000 | 0.0000 |
| 0.0000 | 0.0000 | 0.0000 | 0.0000 | 0.0000 | 0.0000 | 0.0000 | 0.0000 | 0.0000 |

| 65 | 66 | 67 | 68 | 69 | 70 | 71 | 72 | 73 |
|---|---|---|---|---|---|---|---|---|
| 0.0000 | 0.0000 | 0.0000 | 0.0000 | 0.0000 | 0.0000 | 0.0000 | 0.0000 | 0.0000 |
| 0.0000 | 0.0000 | 0.0000 | 0.0000 | 0.0000 | 0.0000 | 0.0000 | 0.0000 | 0.0000 |

| 74 | 75 | 76 | 77 | 78 | 79 | 80 | 81 | 82 |
|---|---|---|---|---|---|---|---|---|
| 0.0000 | 0.0000 | 0.0000 | 0.0000 | 0.0000 | 0.0000 | 0.0000 | 0.0000 | 0.0000 |
| 0.0000 | 0.0000 | 0.0000 | 0.0000 | 0.0000 | 0.0000 | 0.0000 | 0.0000 | 0.0000 |

| 83 | 84 | 85 | 86 | 87 | 88 | 89 | 90 | 91 |
|---|---|---|---|---|---|---|---|---|
| 0.0000 | 0.0000 | 0.0000 | 0.0000 | 0.0000 | 0.0000 | 0.0000 | 0.0000 | 0.0000 |
| 0.0000 | 0.0000 | 0.0000 | 0.0000 | 0.0000 | 0.0000 | 0.0000 | 0.0000 | 0.0000 |

| 92 | 93 | 94 | 95 | 96 | 97 | 98 | 99 | 100 |
|---|---|---|---|---|---|---|---|---|
| 0.0000 | 0.0000 | 0.0000 | 0.0000 | 0.0000 | 0.0000 | 0.0000 | 0.0000 | 0.0000 |
| 0.0000 | 0.0000 | 0.0000 | 0.0000 | 0.0000 | 0.0000 | 0.0000 | 0.0000 | 0.0000 |

| 101 | 102 | 103 | 104 | 105 | 106 | 107 | 108 | 109 |
|---|---|---|---|---|---|---|---|---|
| 0.0000 | 0.0000 | 0.0000 | 0.0000 | 0.0000 | 0.0000 | 0.0000 | 0.0000 | 0.0000 |
| 0.0000 | 0.0000 | 0.0000 | 0.0000 | 0.0000 | 0.0000 | 0.0000 | 0.0000 | 0.0000 |

| 110 | 111 | 112 | 113 | 114 | 115 | 116 | 117 | 118 |
|---|---|---|---|---|---|---|---|---|
| 0.0000 | 0.0010 | 0.0571 | 0.0620 | 0.0102 | 0.0035 | 0.0000 | 0.0010 | 0.0000 |
| 0.0000 | 0.0000 | 0.0000 | 0.0000 | 0.0000 | 0.0000 | 0.0000 | 0.0000 | 0.0000 |

| 119 | 120 | 121 | 122 | 123 | 124 | 125 | 126 | 127 |
|---|---|---|---|---|---|---|---|---|

| | | | | | | | | |
|---|---|---|---|---|---|---|---|---|
| 0.0000 | 0.0000 | 0.0000 | 0.0000 | 0.0000 | 0.0000 | 0.0000 | 0.0000 | 0.0000 |
| 0.0000 | 0.0000 | 0.0000 | 0.0000 | 0.0000 | 0.0000 | 0.0000 | 0.0000 | 0.0000 |

| | | | | | | | | |
|---|---|---|---|---|---|---|---|---|
| 128 | 129 | 130 | 131 | 132 | 133 | 134 | 135 | 136 |
| 0.0000 | 0.0000 | 0.0000 | 0.0000 | 0.0000 | 0.0000 | 0.0000 | 0.0000 | 0.0000 |
| 0.0000 | 0.0000 | 0.0000 | 0.0000 | 0.0000 | 0.0000 | 0.0000 | 0.0000 | 0.0000 |

| | | | | | | | | |
|---|---|---|---|---|---|---|---|---|
| 137 | 138 | 139 | 140 | 141 | 142 | 143 | 144 | 145 |
| 0.0000 | 0.0000 | 0.0000 | 0.0000 | 0.0000 | 0.0000 | 0.0000 | 0.0000 | 0.0000 |
| 0.0000 | 0.0000 | 0.0000 | 0.0000 | 0.0000 | 0.0000 | 0.0000 | 0.0000 | 0.0000 |

| | | | | | | | | |
|---|---|---|---|---|---|---|---|---|
| 146 | 147 | 148 | 149 | 150 | 151 | 152 | 153 | 154 |
| 0.0000 | 0.0000 | 0.0000 | 0.0000 | 0.0000 | 0.0000 | 0.0000 | 0.0000 | 0.0000 |
| 0.0000 | 0.0000 | 0.0000 | 0.0000 | 0.0000 | 0.0000 | 0.0000 | 0.0000 | 0.0000 |

| | | | | | | | | |
|---|---|---|---|---|---|---|---|---|
| 155 | 156 | 157 | 158 | 159 | 160 | 161 | 162 | 163 |
| 0.0000 | 0.0000 | 0.0000 | 0.0000 | 0.0000 | 0.0000 | 0.0000 | 0.0000 | 0.0000 |
| 0.0000 | 0.0000 | 0.0000 | 0.0000 | 0.0000 | 0.0000 | 0.0000 | 0.0000 | 0.0000 |

| | | | | | | | | |
|---|---|---|---|---|---|---|---|---|
| 164 | 165 | 166 | 167 | 168 | 169 | 170 | 171 | 172 |
| 0.0000 | 0.0000 | 0.0000 | 0.0000 | 0.0000 | 0.0000 | 0.0000 | 0.0000 | 0.0000 |
| 0.0000 | 0.0000 | 0.0000 | 0.0000 | 0.0000 | 0.0000 | 0.0000 | 0.0000 | 0.0000 |

| | | | | | | | | |
|---|---|---|---|---|---|---|---|---|
| 173 | 174 | 175 | 176 | 177 | 178 | 179 | 180 | 181 |
| 0.0000 | 0.0000 | 0.0000 | 0.0000 | 0.0000 | 0.0000 | 0.0000 | 0.0000 | 0.0000 |
| 0.0000 | 0.0000 | 0.0000 | 0.0000 | 0.0000 | 0.0000 | 0.0000 | 0.0000 | 0.0000 |

| | | | | | | | | |
|---|---|---|---|---|---|---|---|---|
| 182 | 183 | 184 | 185 | 186 | 187 | 188 | 189 | 190 |
| 0.0000 | 0.0000 | 0.0000 | 0.0000 | 0.0000 | 0.0000 | 0.0008 | 0.0038 | 0.0083 |
| 0.0000 | 0.0000 | 0.0000 | 0.0000 | 0.0000 | 0.0000 | 0.0000 | 0.0000 | 0.0000 |

| | | | | | | | | |
|---|---|---|---|---|---|---|---|---|
| 191 | 192 | 193 | 194 | 195 | 196 | 197 | 198 | 199 |
| 0.0356 | 0.0204 | 0.0047 | 0.0119 | 0.1224 | 0.0747 | 0.0111 | 0.0821 | 0.0368 |
| 0.0000 | 0.0002 | 0.0010 | 0.0050 | 0.2029 | 0.1493 | 0.0123 | 0.0109 | 0.0001 |

| | | | | | | | | |
|---|---|---|---|---|---|---|---|---|
| 200 | 201 | 202 | 203 | 204 | 205 | 206 | 207 | 208 |
| 0.0028 | 0.0269 | 0.0000 | 0.0000 | 0.0101 | 0.0000 | 0.0198 | 0.0279 | 0.0239 |
| 0.0000 | 0.0000 | 0.0000 | 0.0000 | 0.0000 | 0.0000 | 0.0000 | 0.0000 | 0.0000 |

| | | | | | | | | |
|---|---|---|---|---|---|---|---|---|
| 209 | 210 | 211 | 212 | 213 | 214 | 215 | 216 | 217 |
| 0.0209 | 0.0111 | 0.0114 | 0.0026 | 0.0001 | 0.0011 | 0.0000 | 0.0000 | 0.0000 |
| 0.0000 | 0.0000 | 0.0000 | 0.0000 | 0.0000 | 0.0000 | 0.0000 | 0.0000 | 0.0000 |

| | | | | | | | | |
|---|---|---|---|---|---|---|---|---|
| 218 | 219 | 220 | 221 | 222 | 223 | 224 | 225 | 226 |
| 0.0000 | 0.0000 | 0.0000 | 0.0000 | 0.0000 | 0.0000 | 0.0000 | 0.0000 | 0.0000 |
| 0.0000 | 0.0000 | 0.0000 | 0.0000 | 0.0000 | 0.0000 | 0.0000 | 0.0000 | 0.0000 |

| | | | | | | | | |
|---|---|---|---|---|---|---|---|---|
| 227 | 228 | 229 | 230 | 231 | 232 | 233 | 234 | 235 |
| 0.0000 | 0.0000 | 0.0000 | 0.0000 | 0.0000 | 0.0000 | 0.0000 | 0.0000 | 0.0000 |
| 0.0000 | 0.0000 | 0.0000 | 0.0000 | 0.0000 | 0.0000 | 0.0000 | 0.0000 | 0.0000 |

| | | | | | | | | |
|---|---|---|---|---|---|---|---|---|
| 236 | 237 | 238 | 239 | 240 | 241 | 242 | 243 | 244 |
| 0.0000 | 0.0000 | 0.0000 | 0.0000 | 0.0000 | 0.0000 | 0.0000 | 0.0000 | 0.0000 |
| 0.0000 | 0.0000 | 0.0000 | 0.0000 | 0.0000 | 0.0000 | 0.0000 | 0.0000 | 0.0000 |

| | | | | | | | | |
|---|---|---|---|---|---|---|---|---|
| 245 | 246 | 247 | 248 | 249 | 250 | 251 | 252 | 253 |
| 0.0000 | 0.0000 | 0.0000 | 0.0000 | 0.0000 | 0.0000 | 0.0000 | 0.0000 | 0.0000 |
| 0.0000 | 0.0000 | 0.0000 | 0.0000 | 0.0000 | 0.0000 | 0.0000 | 0.0000 | 0.0000 |

| | | | | | | | | |
|---|---|---|---|---|---|---|---|---|
| 254 | 255 | 256 | 257 | 258 | 259 | 260 | 261 | 262 |
| 0.0000 | 0.0000 | 0.0000 | 0.0000 | 0.0000 | 0.0000 | 0.0000 | 0.0000 | 0.0000 |
| 0.0000 | 0.0000 | 0.0000 | 0.0000 | 0.0000 | 0.0000 | 0.0000 | 0.0000 | 0.0000 |

| | | | | | | | | |
|---|---|---|---|---|---|---|---|---|
| 263 | 264 | 265 | 266 | 267 | 268 | 269 | 270 | 271 |
| 0.0000 | 0.0000 | 0.0000 | 0.0000 | 0.0000 | 0.0000 | 0.0000 | 0.0000 | 0.0000 |
| 0.0000 | 0.0000 | 0.0000 | 0.0000 | 0.0000 | 0.0000 | 0.0000 | 0.0000 | 0.0000 |

| | | | | | | | | |
|---|---|---|---|---|---|---|---|---|
| 272 | 273 | 274 | 275 | 276 | 277 | 278 | 279 | 280 |
| 0.0000 | 0.0000 | 0.0000 | 0.0000 | 0.0000 | 0.0000 | 0.0000 | 0.0000 | 0.0000 |
| 0.0000 | 0.0000 | 0.0000 | 0.0000 | 0.0000 | 0.0000 | 0.0000 | 0.0000 | 0.0000 |

| | | | | | | | | |
|---|---|---|---|---|---|---|---|---|
| 281 | 282 | 283 | 284 | 285 | 286 | 287 | 288 | 289 |
| 0.0000 | 0.0000 | 0.0000 | 0.0000 | 0.0000 | 0.0000 | 0.0000 | 0.0000 | 0.0000 |
| 0.0000 | 0.0000 | 0.0000 | 0.0000 | 0.0000 | 0.0000 | 0.0000 | 0.0000 | 0.0000 |

| | | | | | | | | |
|---|---|---|---|---|---|---|---|---|
| 290 | 291 | 292 | 293 | 294 | 295 | 296 | 297 | 298 |
| 0.0000 | 0.0000 | 0.0000 | 0.0002 | 0.0001 | 0.0011 | 0.0291 | 0.0032 | 0.0004 |
| 0.0000 | 0.0000 | 0.0000 | 0.0000 | 0.0000 | 0.0000 | 0.0000 | 0.0000 | 0.0000 |

| | | | | | | | | |
|---|---|---|---|---|---|---|---|---|
| 299 | 300 | 301 | 302 | 303 | 304 | 305 | 306 | 307 |
| 0.0207 | 0.0626 | 0.0050 | 0.0032 | 0.0000 | 0.0004 | 0.0000 | 0.0000 | 0.0000 |
| 0.0000 | 0.0186 | 0.0036 | 0.0011 | 0.0000 | 0.0000 | 0.0000 | 0.0000 | 0.0000 |

| | | | | | | | | |
|---|---|---|---|---|---|---|---|---|
| 308 | 309 | 310 | 311 | 312 | 313 | 314 | 315 | 316 |

| | | | | | | | | |
|---|---|---|---|---|---|---|---|---|
| 0.0000 | 0.0000 | 0.0000 | 0.0000 | 0.0000 | 0.0000 | 0.0000 | 0.0000 | 0.0000 |
| 0.0000 | 0.0000 | 0.0000 | 0.0000 | 0.0000 | 0.0000 | 0.0000 | 0.0000 | 0.0000 |

| 317 | 318 | 319 | 320 | 321 | 322 | 323 | 324 | 325 |
|---|---|---|---|---|---|---|---|---|
| 0.0000 | 0.0000 | 0.0000 | 0.0000 | 0.0000 | 0.0000 | 0.0000 | 0.0000 | 0.0000 |
| 0.0000 | 0.0000 | 0.0000 | 0.0000 | 0.0000 | 0.0000 | 0.0000 | 0.0000 | 0.0000 |

| 326 | 327 | 328 | 329 | 330 | 331 | 332 | 333 | 334 |
|---|---|---|---|---|---|---|---|---|
| 0.0000 | 0.0000 | 0.0000 | 0.0000 | 0.0000 | 0.0000 | 0.0000 | 0.0000 | 0.0000 |
| 0.0000 | 0.0000 | 0.0000 | 0.0000 | 0.0000 | 0.0000 | 0.0000 | 0.0000 | 0.0000 |

| 335 | 336 | 337 | 338 | 339 | 340 | 341 | 342 | 343 |
|---|---|---|---|---|---|---|---|---|
| 0.0000 | 0.0000 | 0.0000 | 0.0000 | 0.0000 | 0.0000 | 0.0000 | 0.0000 | 0.0000 |
| 0.0000 | 0.0000 | 0.0000 | 0.0000 | 0.0000 | 0.0000 | 0.0000 | 0.0000 | 0.0000 |

**Table S2.** List of the contact probabilities with Xenon for each individual amino acid of NPY in the MD simulation of the NPY-bound state of Y2R.

| NPY amino acid | 1 | 2 | 3 | 4 | 5 | 6 | 7 |
|---|---|---|---|---|---|---|---|
| NPY-bound Y2R | 0.0001 | 0.0000 | 0.0001 | 0.0042 | 0.0003 | 0.0018 | 0.2453 |

| 8 | 9 | 10 | 11 | 12 | 13 | 14 | 15 | 16 |
|---|---|---|---|---|---|---|---|---|
| 0.2504 | 0.2527 | 0.3517 | 0.0989 | 0.2847 | 0.0253 | 0.3891 | 0.0205 | 0.0000 |

| 17 | 18 | 19 | 20 | 21 | 22 | 23 | 24 | 25 |
|---|---|---|---|---|---|---|---|---|
| 0.6526 | 0.2651 | 0.0053 | 0.1848 | 0.4804 | 0.0040 | 0.0000 | 0.0002 | 0.0142 |

| 26 | 27 | 28 | 29 | 30 | 31 | 32 | 33 | 34 |
|---|---|---|---|---|---|---|---|---|
| 0.0001 | 0.0000 | 0.0000 | 0.0000 | 0.0000 | 0.0000 | 0.0000 | 0.0000 | 0.0000 |

| 35 | 36 |
|---|---|
| 0.0000 | 0.0000 |
